# A comparison of aspiration thrombectomy with catheter-directed thrombolysis in the management of Paget-Schroetter syndrome

**DOI:** 10.1016/j.jvsv.2026.102637

**Published:** 2026-10-09

**Authors:** Lara Sak, Thomas Munro, Jesus Ulloa, Hugh A. Gelabert

**Affiliations:** a David Geffen School of Medicine at UCLA, Los Angeles, CA; b University of California Los Angeles, Los Angeles, CA; c Division of Vascular and Endovascular Surgery, Department of Surgery, University of California Los Angeles, Los Angeles, CA

**Keywords:** Upper extremity deep vein thrombosis, Thoracic outlet syndrome, Thrombectomy, Thrombolytic therapy

## Abstract

**Objective:**

Acute subclavian vein thrombosis secondary to thoracic outlet syndrome (Paget-Schroetter syndrome [PSS]) requires the prompt clearing of thrombus from the subclavian vein through catheter-directed thrombolysis (CDT) or aspiration thrombectomy (ASP). Our goal is to systematically compare the outcomes of CDT and ASP in the management of PSS.

**Methods:**

Consecutive patients with PSS at a single institution from 2020 through 2025 were reviewed for management with CDT or ASP. Venograms were assessed for patency, stenosis, and subclavian post-thrombotic changes (PTC). PTC were graded based on the degree of luminal encroachment and vessel irregularity: mild (<10%), moderate (10%-50%), and severe (50%-99%). Interventions were assessed: (1) within 1 week of the procedure, (2) before subclavian vein decompression, (3) 2 weeks after subclavian vein decompression, and (4) patency on the last venogram. Standardized outcomes were assessed.

**Results:**

A total of 110 patients with PSS were treated over 5 years. Of these patients, 54 met the inclusion criteria: 28 underwent CDT and 26 underwent ASP. At 1 week following the procedure, rethrombosis occurred in 1 (3.6%) patient who underwent CDT and 6 (23.1%) patients who underwent ASP (*P* < .5). Failure to clear thrombosis occurred in 13.8% of patients who underwent ASP. The composite rate of early thrombosis and procedural failure was higher in the ASP cohort. Before PSS surgery, occlusion was noted in 1 (3.6%) patient who underwent CDT and 8 (30.8%) patients who underwent ASP (*P* < .05). PTC was mild in 13 (46.4%) patients receiving CDT and 5 (19.2%) receiving ASP (*P* < .05); moderate in 5 (17.9%) receiving CDT and 5 (19.2%) receiving ASP; and severe in 9 (32.1%) receiving CDT and 8 (30.8) receiving ASP. At early follow-up after PSS surgery, PTC was mild in 10 (43.5%) patients receiving CDT and 4 (23.5) receiving ASP; moderate in 2 (8.7%) receiving CDT and 1 (5.9%) receiving ASP; and severe in 9 (39.1%) receiving CDT and 6 (35.3%) receiving ASP. Occlusion was noted in 2 (8.7%) patients receiving CDT and 6 (35%) receiving ASP. Recanalization resulted in final patency in 22 (95%) patients receiving CDT and 15 (88%) receiving ASP.

**Conclusions:**

Both ASP and CDT may be employed for clearing thrombus in PSS; however, they differ in early efficacy, PTC, and patency. Our results suggest that while often more rapid and convenient, ASP may be inferior to CAT in the management of PSS.

**Article Highlights**

• **Type of Research:** Single-center retrospective cohort study
• **Key Findings:** In the comparison of aspiration thrombectomy with catheter-directed thrombolysis for the management of acute Paget-Schroetter syndrome, aspiration thrombectomy was associated with higher rates of early rethrombosis (23.1% vs 3.6%) and preoperative occlusion (30.8% vs 3.6%). Final patency was similar between the groups.
• **Take Home Message:** While both aspiration thrombectomy and catheter-directed thrombolysis may be employed for thrombus clearance in the management of acute Paget-Schroetter syndrome, they differ in early efficacy and post-thrombotic changes. Consistent with previous reports, the current results revealed that aspiration thrombectomy was associated with higher rates of early rethrombosis and occlusion, requiring repeat intervention.

Paget-Schroetter syndrome (PSS), also known as effort thrombosis, is acute subclavian vein (SCV) thrombosis due to venous thoracic outlet syndrome. Extrinsic compression of the SCV at the thoracic outlet leads to endothelial injury, fibrosis, and resultant thrombosis.^1^ PSS is a rare condition with an estimated incidence of 1 to 2 cases per 100,000 individuals per year.^2^, ^3^ It commonly affects young, otherwise healthy individuals and athletes, and it presents with the sudden onset of pain and swelling in the affected upper extremity.^2^ The prompt and effective treatment of PSS is critical for preventing chronic venous obstruction and long-term morbidity and disability.^4^

The optimal management of PSS has evolved. Historically, its treatment consisted of anticoagulation alone.^5^ However, anticoagulation alone is associated with significant rates of symptom recurrence and chronic disability.^6^, ^7^, ^8^ Recognition of these suboptimal outcomes prompted a shift away from conservative management toward more aggressive interventions aimed at the early restoration of venous patency and the relief of the underlying mechanical compression.^7^, ^9^, ^10^ As a result, the current management of acute PSS typically consists of anticoagulation and prompt thrombus removal within 2 weeks of thrombosis, followed by surgical decompression to address the underlying pathology.^11^

Thrombus clearance is commonly achieved using endovascular techniques such as thrombolysis and mechanical thrombectomy.^12^ Catheter-directed thrombolysis (CDT) involves the local infusion of a thrombolytic agent via a catheter into the thrombosed vessel. The use of CDT for the management of acute PSS has led to improved symptom resolution and venous patency compared with anticoagulation alone.^13^ Alternatively, aspiration thrombectomy (ASP) is a newer technique that utilizes a catheter with suction to rapidly remove a thrombus within a vessel without the use of thrombolytic agents.^14^ While both ASP and CDT may be employed for thrombus clearance, CDT is the most common endovascular therapy for the management of acute PSS, whereas ASP is rarely used as a standalone treatment and is generally used as an adjunct to thrombolysis.^15^ Consequently, data comparing the outcomes of ASP and CDT in the management of PSS are limited. The objective of this study is to systematically compare the outcomes of ASP and CDT as standalone treatments in the management of acute PSS.

## Methods

We conducted a retrospective review of consecutive patients with PSS presenting at our institution from 2020 through 2025. Patients with PSS who underwent initial management with CDT or ASP with available pre- and postoperative imaging were reviewed. Patients were excluded from the study if they presented with a subacute or chronic presentation (beyond the 2-week window for thrombolysis) or if they were managed with a combination of techniques, including both CDT and ASP. Patients who were lost to follow-up were also excluded. Data were derived from the review of medical charts and venogram imaging. Venograms were used for the diagnosis of PSS and the serial evaluation of the SCV. The variables collected included age, sex, hand dominance, athletic status, pulmonary embolism, coagulopathies, deep vein thrombosis (DVT) location, initial management, intervals between initial presentation and subsequent interventions, and follow-up period. Standardized functional outcomes were assessed at each visit using the Somatic Pain Scale (SPS) and Quick Disability of Arm, Shoulder, and Hand (QuickDASH). This research project was conducted under an institutional review board-approved protocol.

### Treatment protocol

Venograms were obtained at the initial presentation. The decision for ASP or CDT was made by the treating physician at the time of intervention. Data on the catheter manufacturer and size of aspiration catheters were unavailable. Intravascular ultrasound scans were not routinely conducted after ASP or CDT. Patients were placed on anticoagulation following the initial procedure and then referred to the vascular surgery service for PSS evaluation. All patients receiving heparin were managed using pharmacy protocol that adjusted the dose to the target partial thromboplastin time. Those who elected to proceed with surgical decompression were evaluated with a diagnostic venogram at the time of surgery. A follow-up venogram was scheduled approximately 2 weeks after surgery to assess the condition of the SCV. No stents were used after ASP/CDT (and before the first rib resection) or after surgical decompression. Compression sleeves were not routinely used for upper extremities following thrombolysis.

### Venogram assessments

Venograms were assessed for patency, stenosis, and post-thrombotic changes (PTC) in the SCV. Interventions were assessed at four time points: (1) within 1 week following the declot procedure (early procedural success), (2) immediately before SCV decompression surgery (preop assessment), (3) 2 weeks following SCV decompression surgery (postop assessment), and (4) final patency on the last venogram (final patency). PTC were graded based on the degree of luminal encroachment and vessel irregularity as mild (<10%), moderate (10%-50%), and severe (50%-99%). Occlusion was defined on venography as the absence of contrast flow in a segment of the SCV.

### Statistical analysis

Statistical analysis was performed using the Fisher exact test for categorical variables, Student *t*-test for continuous variables, and Wilcoxon signed-rank test for repeated measurements. Statistical significance was established at *P* < .05.

## Results

A total of 110 consecutive patients were treated at our institution for PSS from January 1, 2020, through August 1, 2025. Two patients were excluded for undergoing management with both ASP and CDT. Fifty-four patients met the inclusion criteria: 26 who underwent ASP and 28 who underwent CDT. The study sample included 33 men and 21 women. Of these, 10 were athletes. Patients treated with ASP were older than those treated with CDT (39.5 vs 31.1; *P* = .009). All other baseline demographics were similar between the groups (Table I).

**Table I:** Demographics

	Total (n = 54)	ASP (n = 26)	CDT (n = 28)
Age, years average	35.2	39.5^a^	31.1^a^
Female, n (%)	21	11 (42)	10 (36)
Athletes, n (%)	10	3 (12)	7 (25)
Coagulation abnormalities, n (%)	3	2 (8)	1 (4)
Pulmonary embolism, n (%)	13	7 (27)	6 (21)
Right-handed, n (%)	42	21 (81)	21 (75)
DVT (right), n (%)	27	11 (42)	16 (57)
DVT (left), n (%)	27	15 (58)	12 (43)

*ASP*, Aspiration thrombectomy; *CDT*, catheter-directed thrombolysis; *DVT*, deep vein thrombosis.

a *P* < .05.

No statistically significant differences were noted between the ASP and CDT groups in any of the measured time intervals. The mean time interval from DVT presentation to the declot procedure was 3 days for the ASP group and 4.9 days for the CDT group. The mean time interval from the declot procedure to surgical decompression was 153.8 days for the ASP group and 106 days for the CDT group. The mean duration from surgical decompression to the most recent follow-up was 205.6 days for the ASP group and 200.5 for the CDT group (Table II).

**Table II:** Time intervals

Time, days	All	ASP (n = 26)	CDT (n = 28)	*P* value
DVT to declot	4	3	4.9	.221
Declot to consult	69.8	104.7	37.3	.146
Declot to initial venogram	131.5	168.3	96.2	.170
Declot to surgery	128.5	153.8	106	.337
Declot to postop venogram	119.9	126.1	114.9	.678
Consult to initial venogram	58.6	60.5	56.7	.803
Surgery to postop venogram	25.5	26.4	24.7	.807
Surgery to final follow-up	202.9	205.6	200.5	.972

*ASP*, Aspiration thrombectomy; *CDT*, catheter-directed thrombolysis.

### Early procedural success

Within 1 week following the declot procedure, rethrombosis occurred in 1 (3.6%) patient receiving CDT and 6 (23.1%) patients receiving ASP (*P* < .05). Failure to clear thrombosis occurred in 1 (3.8%) patient receiving ASP. The combined end point of early rethrombosis and failure was noted in 1 (3.6%) patient receiving CDT and 7 (26.9%) patients receiving ASP (*P* < .05). Reintervention was required to restore patency after the declot procedure in 6 (23%) patients receiving ASP and 1 (3%) receiving CDT (Table III).

**Table III:** Assessment of procedural success after 1 week

	ERT	%	FAIL	%	ERT/FAIL	%
ASP	6^a^	23	1	4	7^a^	27
CDT	1^a^	4	0	0	1^a^	4

*ASP*, Aspiration thrombectomy; *CDT*, catheter-directed thrombolysis; *ERT*, early rethrombosis of subclavian vein within 1 week of declot; *ERT/FAIL*, combined end point; *FAIL*, procedural failure to declot subclavian vein.

a *P* < .05.

### Preop assessment

In the evaluation before PSS surgery, occlusion was noted in 1 (3.6%) patient receiving CDT and 8 (30.8%) receiving ASP (*P* < .05). PTC was assessed as mild in 13 (46.4%) patients receiving CDT and 5 (19.2%) receiving ASP (*P* < .05); moderate in 5 (17.9%) receiving CDT and 5 (19.2%) receiving ASP; and severe in 9 (32.1%) receiving CDT and 8 (30.8) receiving ASP (Table IV).

**Table IV:** Assessment of post-thrombotic changes

	Mild	%	Moderate	%	Severe	%	Occlusion	%
Before surgical decompression (preop interval assessment)								
ASP	5^a^	19	5	19	8	31	8^a^	31
CDT	13^a^	46	5	18	9	32	1^a^	4
Following surgical decompression (postop assessment)								
ASP	4	24	1	6	6	35	6	35
CDT	10	44	2	9	9	39	2	9

*ASP*, Aspiration thrombectomy; *CDT*, catheter-directed thrombolysis.

a *P* < .05.

### Postop assessment

At early follow-up after PSS surgery, PTC was assessed as mild in 10 (43.5%) patients receiving CDT and 4 (23.5) receiving ASP; moderate in 2 (8.7%) receiving CDT and 1 (5.9%) receiving ASP; and severe in 9 (39.1%) receiving CDT and 6 (35.3%) receiving ASP. Occlusion was noted in 2 (8.7%) patients receiving CDT and 6 (35%) receiving ASP (Table IV).

### Final patency

Recanalization resulted in final patency in 22 (95%) patients receiving CDT and 15 (88%) receiving ASP (Table V).

**Table V:** Final patency

	Patent	%	Occlusion	%
ASP	15	88	2	12
CDT	22	96	1	4

*ASP*, Aspiration thrombectomy; *CDT*, catheter-directed thrombolysis.

### Standardized outcomes

At initial presentation, the mean SPS scores were 1.3 in the CDT group and 2.3 in the ASP group, with the corresponding mean QuickDASH scores of 31.4 and 28.8. At final follow-up, the mean SPS scores were 0.3 in the CDT group and 0.2 in the ASP group, with the corresponding mean QuickDASH scores of 6.1 and 16. Within-group analysis revealed a significant improvement in QuickDASH scores in the CDT group, which decreased from a mean of 31.4 at initial presentation to 6.1 at final follow-up (*P* = .00036). No other standardized measure analysis demonstrated statistical significance (Table VI).

**Table VI:** Standardized outcomes

	All	ASP (n = 26)	CDT (n = 28)
Pain (initial, mean)	1.8	2.3	1.3
Pain (final, mean)	0.2	0.2	0.3
QuickDASH (initial, mean)	30.1	28.8	31.4^a^
QuickDASH (final, mean)	10.1	16	6.1^a^

*ASP*, Aspiration thrombectomy; *CDT*, catheter-directed thrombolysis; *QuickDASH*, Quick Disability of Arm, Shoulder, and Hand.

a *P* < .05.

## Discussion

The management of acute PSS has evolved from anticoagulation alone to early thrombus removal with thrombolysis and, more recently, ASP. Early reports in the late 1970s and 1980s described the use of thrombolysis for acute PSS in response to the poor outcomes associated with anticoagulation alone.^2^, ^16^, ^17^ Subsequent studies have since demonstrated that thrombolysis in the initial 2-week period following acute thrombosis provides significant benefits for symptom resolution and restoration of patency, resulting in its widespread use in the current management of PSS.^10^, ^11^, ^13^ Because thrombus clearance alone does not address the underlying compression, evidence suggests that optimal outcomes are achieved when thrombolysis is followed by surgical decompression of the thoracic outlet.^7^, ^18^, ^19^

To reduce bleeding complications, localized thrombolysis with CDT is recommended over systemic thrombolysis.^20^ However, despite its advantages, CDT remains associated with increased bleeding risk, prolonged treatment time, the need for intensive care monitoring, and increased cost.^21^ These limitations have led to the increasing interest in alternative methods of thrombus clearance, such as ASP. ASP has recently emerged as a promising alternative method for thrombus clearance in acute PSS, especially when thrombolysis is contraindicated.^22^ Unlike CDT, ASP relies on vacuum suction for the mechanical extraction of a clot, avoiding the need for thrombolytic agents. ASP enables rapid clot removal in a single session. In addition to minimizing bleeding risk, ASP reduces the procedure time, cost, and the need for multiple treatment sessions compared with CDT and other endovascular techniques.^23^, ^24^ However, despite increasing reports of ASP use for PSS, data on the use of ASP as a standalone treatment are limited, with most studies describing its role as an adjunct to thrombolysis, either as a part of pharmaco-mechanical thrombectomy or in combination with CDT.^25^, ^26^, ^27^, ^28^ Furthermore, direct comparative studies of ASP and CDT for PSS are limited and often focus on cost and short-term efficacy, with limited follow-up, highlighting the need for comparative studies evaluating clinical outcomes.^23^

In this single-center retrospective study, we identified 26 patients with PSS who underwent ASP for the initial management of DVT and compared them with 28 patients who received CDT as the initial treatment. We evaluated several outcomes, including thrombus clearance, early rethrombosis, PTC, and patency across different time points. We observed that ASP was associated with statistically significant higher rates of early rethrombosis and a higher composite rate of early rethrombosis and procedural failure. Pre- and postoperative evaluation revealed a higher incidence of mild PTC with CDT and more frequent venous occlusion in the ASP group, although final patency was similar between the groups. Regarding standardized functional outcomes, the CDT group showed significant improvement in QuickDASH scores from presentation to final follow-up.

Compared with CDT, ASP was associated with significantly higher rates of early rethrombosis and an increase in procedural failure, although the latter did not reach significance. The combined end point of early rethrombosis and procedural failure was also significantly more frequent with ASP, suggesting that ASP may have lower early efficacy than CDT. While the data on the use of ASP for PSS are limited, our findings are consistent with those of other studies that have noted early failure when ASP is used alone for thrombus clearance, with several studies reporting the need for additional declotting techniques following ASP.^29^, ^30^, ^31^ Fuller et al^23^ reported that 70% of patients with PSS who underwent initial management with ASP also required the use of CDT. Similarly, Teter et al^24^ reported that 69% of patients with PSS treated with ASP required adjunctive treatment with CDT, suggesting that ASP may not be as effective as a standalone treatment for initial thrombus clearance. The higher rate of early rethrombosis with ASP may reflect incomplete thrombus removal, as many studies have defined treatment with ASP successful if at least 70% of the thrombus is removed.^14^, ^23^, ^32^, ^33^

PTC were noted in most patients following declotting; these ranged from mild to severe and occlusion. Our data showed that both pre- and postoperatively, CDT was associated with mild PTC, whereas ASP led to higher rates of venous occlusion. However, neither difference reached statistical significance in the postoperative period. The higher incidence of occlusion with ASP may be due to incomplete thrombus clearance or endothelial damage resulting from mechanical trauma during aspiration. Notably, severe PTC was observed in more than one-third of patients in both groups in the postoperative assessment. This observation may reflect the prolonged interval to surgical decompression in our cohort and the early timing of the postoperative venogram, which preceded subsequent venoplasty.

PTC, characterized by venous wall fibrosis with variable degrees of luminal narrowing, is frequently observed after DVT and is most commonly discussed in the broader context of post-thrombotic syndrome (PTS).^34^, ^35^ While PTC reflects structural abnormalities, PTS represents a clinical sequela characterized by symptoms such as pain, swelling, and functional impairment, with a reported incidence of 7% to 46% following upper-extremity DVT.^36^, ^37^ Given the significant morbidity associated with PTS, reducing its incidence is a primary objective of DVT treatment, with interventions promoting early and complete thrombus removal and the maintenance of patency to potentially reduce risk.^38^, ^39^ Interestingly, our finding of more severe PTC following ASP in the upper extremity contrasts with data from lower-extremity DVT, in which PTS has been reported less frequently after pharmaco-mechanical thrombectomy, including aspiration-based techniques, compared with CDT.^40^, ^41^ This discrepancy may be attributable to the fundamental differences in the upper- and lower-extremity venous systems, with the upper-extremity venous system being characterized by a smaller vessel diameter, lower blood flow, and lower pressure.^42^, ^43^, ^44^

Despite differences in early rethrombosis and venous occlusion, we observed no significant difference in final patency between the CDT and ASP groups, suggesting that subsequent interventions, such as thoracic outlet decompression and venoplasty, may facilitate the restoration of long-term patency. Evaluation of standardized functional outcomes in the CDT group revealed a significant improvement in QuickDASH scores from initial presentation to final follow-up. This finding suggests that CDT may be associated with improved functional outcomes.

Our study has several limitations. Due to the retrospective design, there is concern for selection bias, as the choice between ASP and CDT was made at the discretion of the treating physician. This may have resulted in unmeasured confounding elements related to differences in patient characteristics and the severity and extent of DVT. The mean interval from the declot procedure to surgical decompression was longer than is typically recommended, reflecting referral patterns at or institution, which may limit the generalizability of our findings. Additionally, our single-center study has limited statistical power due to the relatively small sample size. Thrombolytic dosing, anticoagulation regimens, and adjunctive venographic interventions varied, which may have influenced both early and long-term outcomes. Additionally, the objective measurement of residual SCV stenosis was not available. Finally, the assumptions underlying our criteria for success are different for CDT and ASP. With CDT, it is a standard practice to plan for repeat sessions to clear thrombus. For ASP, the standard expectation is a single session to clear thrombus. In this instance, we found that a single session of ASP was not as effective as CDT in terms of initial success.

## Conclusions

To the best of our knowledge, this is one of the first studies to systematically compare the outcomes of ASP and CDT with respect to success in declot, PTC, and patency in the management of acute PSS. We observed that while both ASP and CDT may be employed for thrombus clearance from the SCV in PSS, they differ in early efficacy, PTC, and patency. Consistent with previous reports, our findings suggest that ASP may be associated with higher rates of early rethrombosis and occlusion, requiring repeat intervention. We caution that these are preliminary findings based on a limited sample size, and future studies are needed to further investigate the comparative efficacy and long-term outcomes of both approaches.

## Author Contributions

Conception and design: LS, TM, JU, HG

Analysis and interpretation: LS, JU, HG

Data collection: LS, HG

Writing the article: LS, JU, HG

Critical revision of the article: LS, TM, JU, HG

Final approval of the article: LS, TM, JU, HG

Statistical analysis: LS, HG

Obtained funding: Not applicable

Overall responsibility: HG

## Funding

None.

## Disclosures

None.
